# Comparative Transcriptome Analyses of Geriatric Rats Associate Age-Related Erectile Dysfunction With a lncRNA-miRNA-mRNA Regulatory Network

**DOI:** 10.3389/fendo.2022.887486

**Published:** 2022-07-11

**Authors:** Xuan Zhou, Rong Cong, Liangyu Yao, Xiang Zhou, Jiaochen Luan, Qijie Zhang, Xu Zhang, Xiaohan Ren, Tongtong Zhang, Xianghu Meng, Ninghong Song

**Affiliations:** ^1^ Department of Urology, The First Affiliated Hospital of Nanjing Medical University, Nanjing, China; ^2^ Department of Urology, The Affiliated Kizilsu Kirghiz Autonomous Prefecture People’s Hospital of Nanjing Medical University, Artux, China

**Keywords:** lncRNA (long non-coding RNA), erectile dysfunction, aging, bioinformatics, miRNA, RNA sequence analyses

## Abstract

**Background:**

The key regulatory roles of long non-coding RNAs (lncRNAs) in age-related erectile dysfunction (A-ED) are unknown.

**Aim:**

This study aimed to identify putative lncRNAs that regulate age-related erectile dysfunction *via* transcriptome analyses, and to predict their specific regulatory routes *via* bioinformatics methods.

**Methods:**

22 geriatric male SD rats were divided into age-related erectile dysfunction (A-ED) and negative control (NC) groups after evaluations of intracavernous pressure (ICP). By comparative analysis of transcriptomes of cavernosal tissues from both groups, we identified differentially expressed lncRNAs, miRNAs, and mRNAs. Seven differentially expressed lncRNAs were selected and further verified by quantitative real-time polymerase chain reactions (RT-qPCR). The construction of the lncRNA-miRNA-mRNA network, the Gene Ontology (GO) term enrichment, and Kyoto Encyclopedia of Genes and Genomes (KEGG) pathway analysis were performed in Cytoscape.

**Results:**

From comparative transcriptome analyses of A-ED and NC groups, 69, 29, and 364 differentially expressed lncRNAs, miRNAs, and mRNAs were identified respectively. Differentially expressed lncRNAs were culled to seven, which were all verified by qPCR. Three of these lncRNAs (ENSRNOT00000090050, ENSRNOT00000076482, and ENSRNOT00000029245) were used to build regulatory networks, of which only ENSRNOT00000029245 was successful. Moreover, GO and KEGG analyses demonstrated that these lncRNAs possibly regulated muscle myosin complex, muscle cell cellular homeostasis, and ultimately erectile function in rats through PI3K-Akt, fluid shear stress, and atherosclerosis pathways.

**Conclusion:**

Our study identified differentially expressed lncRNAs, miRNAs, and mRNAs through comparisons of transcriptomes of geriatric rats. An identified lncRNA verified by qPCR, was used to construct a lncRNA-miRNA-mRNA regulatory network. LncRNA ENSRNOT00000029245 possibly regulated downstream mRNAs through this regulatory network, leading to apoptosis in the cavernous tissue, fibrosis, and endothelial dysfunction, which ultimately caused ED. These findings provide seminal insights into the molecular biology of aging-related ED, which could spur the development of effective therapeutics.

## Introduction

Erectile dysfunction (ED) is an inability to either achieve or maintain a satisfactory penile erection for sexual activity. It is more common in middle-aged and elderly men, especially those over 40 years of age (1). Moreover, most ED patients have an organic etiology (2). ED is not only associated with aging, but also with cardiovascular and endocrine diseases, and neurogenic illnesses (3).

By 2050, 22% of the global population is predicted to have become over 60 years of age (4), and this will result in more ED patients, whose current prevalence rate is 2% and 86% for men under 40 and over 80 years of age respectively (5). Yet by 2025, 332 million people are predicted to have suffered from ED (6). Oral selective phosphodiesterase type 5 inhibitors (PDE5-Is) are common first-line therapies. They inhibit the conversion of cyclic guanosine monophosphate (cGMP) to cyclic guanosine monophosphate(GMP), leading to relaxation of vascular and cavernosal smooth cells, and this ultimately maintains an erection. Vacuum erection devices (VED), intracavernosal injections (ICI), and penile implants are used as second-line therapies. Nevertheless, they have a variety of shortcomings, resulting in the current unsatisfactory clinical treatment of ED (2). Hence, further investigations on the molecular mechanisms of ED are not only needed but could also lead to the development of effective therapies.

Senescence is a major risk factor for various chronic diseases, including type 2 diabetes mellitus (T2DM), cardiovascular disease, and cancer (7). Senescence is a programmed progression caused by accumulated DNA, protein, and lipid damage (8). Relatedly, endothelial dysfunction, which is closely related to erectile dysfunction, occurs due to aging (9–11). Specifically, accumulated damage due to aging leads to dysfunction of the vascular endothelium, which in turn leads to vasculogenic erectile dysfunction (12).

Long non-coding ribonucleic acids (lncRNAs) are ribonucleic acids (RNA) that are both longer than 200 base pairs and not translated into proteins (13, 14). They play important roles in transcriptional regulation, the formation of subcellular structures, epigenetic gene regulation, and programmed cellular development (15, 16). In cardiovascular diseases, lncRNAs regulate downstream biological functions by positive and negative modulatory effects, playing roles as miRNA sponges and co-expression with miRNAs (17). LncRNAs have been associated with ED (18, 19). For example, in diabetes mellitus-induced erectile dysfunction, lncRNA MALAT1 functioned as a sponge for miR-206 to upregulate the expression of VEGFA, thus boosting bone marrow-derived mesenchymal stem cells (BM-MSCs) differentiation into endothelial cells for rectification of ED (20). However, there exists a paucity of studies on this topic. To fill this research gap, we aimed to elucidate possible key roles of lncRNA in age-related ED (A-ED) by constructing a lncRNA-miRNA-mRNA regulatory network *via* comparative transcriptome analyses.

## Methods

### Animals Procedures

We used 22 geriatric male SD rats (20 months) purchased from the Experimental Animal Center of Nanjing Medical University, and our research was approved by the Animal Care and Use Committee of the First Affiliated Hospital of Nanjing Medical University. The animals were anesthetized with pentobarbital natrium (40 mg/kg) by intraperitoneal injection.

### Erectile Evaluation

After anesthesia, dissections were made along the penis midline to expose the carotid artery. A neck artery was cannulated and a heparinized 25-gauge needle was inserted into the corpora cavernosum of the penis to measure both mean arterial pressure (MAP) and ICP. Electrical stimulation was set at 5 V, 15 Hz, pulse width 0.2ms, and the stimulation lasted for 1 min. The BL-420S Biological Functional System (Chengdu Taimeng Technology Co, LTD, Chengdu, China) was used to measure pressure and generate electrical stimulation. Each experiment was done in triplicate. The ratio of the maximal ICP to the corresponding MAP (ICP/MAP) was calculated to estimate the erectile function. The corpus cavernosum tissue was isolated and stored -80°C for further experiments.

### Masson Trichrome Staining

Experiments were carried out as previously described (21). Penile tissue was fixed with 4% paraformaldehyde and embedded in paraffin. Masson trichrome staining was used to observe, under 200x magnification, then evaluate the ratio of smooth muscle to collagen area in penile tissue.

### Total RNA Isolation and Transcriptome Sequencing (RNA-seq)

The rats were divided into geriatric A-ED (erectile dysfunction) and NC (negative control) groups based on the erectile evaluation results. ICP/MAP > 35% was defined as the geriatric NC group, and ICP/MAP < 35% was defined as the geriatric ED group. After stripping the urethra and other connective tissue from the penis, the cavernous tissue was retained. Cavernous tissue was minced into small pieces with a low-temperature grinder (Servicebio, KZ-III-F) under grinder parameters of 60Hz & -10°C. Then, total RNA was extracted from pieces of cavernous tissues or cells using a TRIzol (Invitrogen, Carlsbad, CA, USA) based protocol. RNA concentration and purity were determined *via* a NanoDrop 2000 spectrophotometer (Madison, WI, USA). Ribosomal RNA (rRNA) was separated from total RNA and RNA integrity was analyzed *via* an Agilent RNA 6000 Nano Kiton an Agilent 2100 Bioanalyzer. Residual total RNA was broken into short fragments for 2 min, then reverse transcribed into cDNA with random hexamers as primers *via* a SuperScript III First-Strand Synthesis protocol (Invitrogen). Synthetized products were purified using a QIAquick PCR Purification Kit (QIAGEN). DNA fragment ends were repaired and ligated with poly (A) tails to connect them to Illumina sequencing adapters. Then, the second strand was digested and the appropriate fragment was identified for PCR amplification. The cDNA sequencing library was made and subsequently sequenced using an Illumina HiSeq TM 4000 platform (Gene Denovo Biotechnology Co., Guangzhou, China). Only reads that met the following criteria were used as raw data (1) reads did not contain adapters; (2) reads did not contain more than 10% of unknown nucleotides (N); (3) reads did not contain more than 50% of low quality (Q-value ≤ 20) bases. These reads were aligned to Ribosome RNA (rRNA) and mapped to a rat reference genome using HISAT2 (22).

### Quantitative Real-Time PCR

We extracted total RNA from previously isolated corpus cavernosum tissues using a Trizol (Invitrogen, USA) based protocol. Then, cDNA was synthesized using HiScript^®^ III All-in-one RT SuperMix Perfect for qPCR (Vazyme, China). Next, we utilized the StepOne Plus Real-Time PCR system (Applied Biosystems, USA) to perform qPCR. Fold changes in mRNA expression were calculated using the 2−ΔΔCt method and normalized against β-actin with ABI Step One software version 2.1. PCR primer sequences were synthesized by TSINGKE Biological Technology (Nanjing, China) and are listed in **Supplementary Table S1**.

### Bioinformatic Analysis

Transcriptome data were normalized and analyzed in R using the edgR package. Then three validated lncRNAs were selected (ENSRNOT00000090050, ENSRNOT00000076482, and ENSRNOT00000029245). For prediction analysis, we defined differently expressed miRNAs and mRNAs as logFC >2 and *p <*0.05. MiRNAs target prediction tools TargetScan, miRcode, and miRDB were used to predict miRNAs targets. All relevant annotations of miRNAs were derived from miRbase.

The network was constructed based on ceRNA theory as previously described (21). (1) Spearman rank correlation coefficient (SCC) was used to evaluate expression correlation between miRNAs-mRNAs or miRNA-lncRNAs. RNA interaction pairs that had SCC<0 were defined as either negatively co-expressed lncRNA-miRNA or mRNA-miRNA pairs. All RNAs were differentially expressed. The mRNA and lncRNA were predicted as miRNA target genes. (2) Pearson correlation coefficient (PCC) was used to assess expression correlations between lncRNA and mRNA. ceRNA interaction pairs that had PCC > 0.5 were defined as co-expressed lncRNA-mRNA pairs. RNA interaction pairs of mRNA and lncRNA were termed as negatively or postively co-expressed with a common miRNA. Pairs of mRNA and lncRNA were targeted negatively or positively co-expressed with a common miRNA. (3) The significance of common miRNA sponges differences between the two genes was tested by a hypergeometric cumulative distribution function test. The filtering condition was *p*< 0.05. Finally, all eligible RNA interaction pairs were collected and combined into a network, which was visualized in Cytoscape 3.8.2 (http://www.cytoscape.org/).

Gene ontology (GO, http://www.geneontology.org/) enrichment analysis and Kyoto Encyclopedia of Genes and Genomes (KEGG, http://www.genome.jp/kegg) pathway analysis of differentially expressed mRNAs were performed using the R package clusterProfiler. An alpha level of *p*<0.05 was used to test statistical significance.

### Cell Culture

The rat vascular endothelium cell line (RAOEC) was purchased from Shanghai Jingkang Biological Co., LTD (Shanghai, China) and cultured at 37 °CC in 5% CO_2_. The endothelial cell lines were characterized by double-immunofluorescent staining for biomarkers CD31 and vWF (Proteintech, China). DMEM medium, supplemented with 10% FBS, and 100 units/mL penicillin, were used in cell culture (Gibco).

### Induction and Evaluation of Senescence

ROAEC cells were treated with hydrogen peroxide (H_2_O_2_) to induce senescence (23, 24). Cells were seeded onto 6-well plates (10^6^/well) in a full growth medium. We choose the optimized concentration (100 µM) to build a cellular aging model according to the references (25). After 24 hours, the cells were treated with H_2_O_2_ (100μM) and DMSO (0.01%) respectively for 24 hours. Senescence-associated β-galactosidase (sa-β-Gal) staining was performed using a β-galactosidase enzyme assay kit (Beyotime, China) between the H_2_O_2_-treated group and the DMSO group.

### Statistical Analysis

All data analyses were done using SPSS software (SPSS V 24.0). T-tests were used to determine the statistical significance of differences between two groups of parametric data whereas Mann-Whitney tests were used for nonparametric data. Statistical significances of GO and KEGG analyses were determined using Fisher’s exact tests. An alpha level of *p*<0.05 was used for all statistical tests.

## Results

### Erectile Function Evaluation

**Figure 1:**
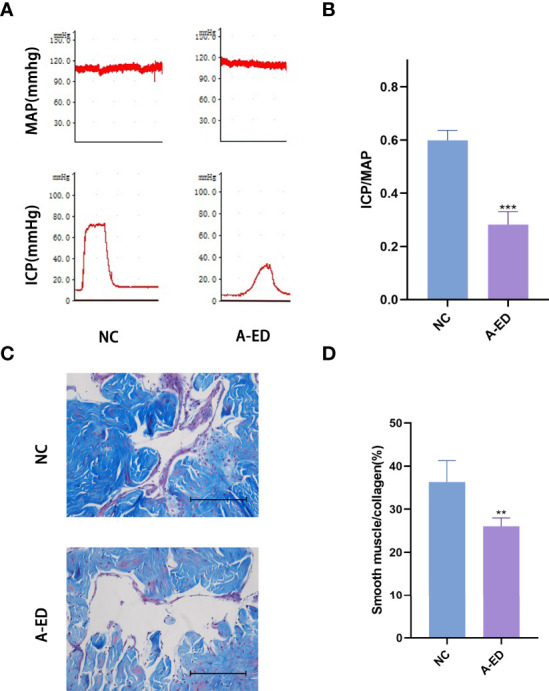
Evaluation of Erectile Dysfunction **(A)**: Mean arterial pressure (MAP) and intracavernous pressure (ICP) of rats in the A-ED group and NC group. **(B)**: Bar plot of the ICP/MAP of A-ED group and NC group. ***p < 0.001. **(C)**: Masson-trichrome (Masson) staining showing the fibrosis in the A-ED and NC groups. **(D)** Bar plot of the ratio of smooth muscle to collagen in the A-ED and NC groups. **p < 0.01.

Among 22 rats, we selected ICP/MAP less than 0.35 as the low-pressure group and ICP/MAP greater than 0.35 as the high-pressure group, and when combined with the results of the Masson staining, 22 rats were divided into A-ED group and NC group (n=7, n=15). There was no significant difference in MAP between the A-ED and NC groups (*p*=0.3777). However, the ratio of ICP/MAP was lower in the A-ED than the NC group (**Figures 1A, B**). Masson trichromatic staining results showed that the smooth muscle/collagen ratio was significantly lower in the A-ED than the NC group (**Figures 1C, D**). Thus, this A-ED rat model was valid for studies on age-related erectile dysfunction.

### Results of RNA-seq Data

**Figure 2:**
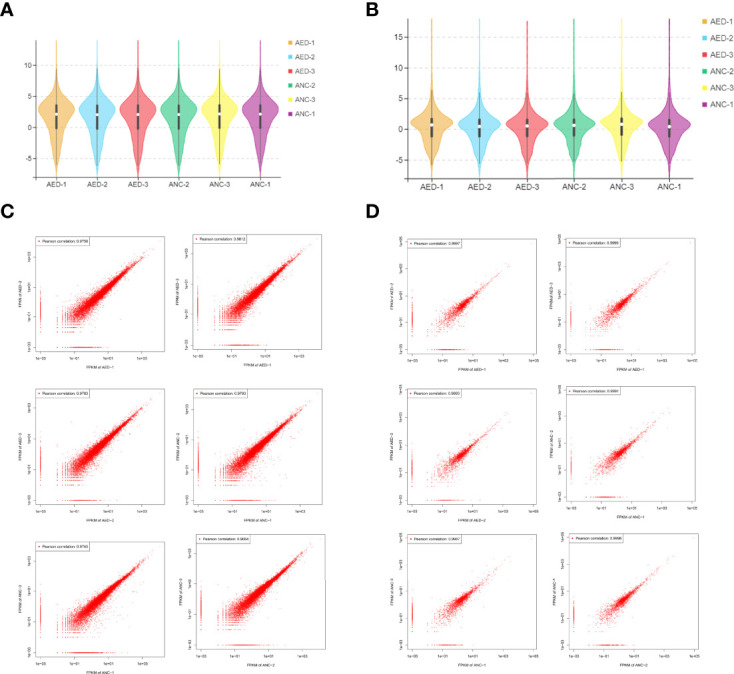
Data distribution and quality assessment. **(A)** A violin diagram of the FPKM value of all mRNAs within each sample. **(B)** A violin diagram of the FPKM value of all lncRNAs within each sample. **(C)** Scatter plot of correlation between mRNAs expression levels in two different samples. **(D)** Scatter plot of correlation between lncRNA expression levels of two different samples.

A total of 5,307 lncRNAs, 1,322 miRNAs, and 22,050 mRNAs were identified by sequencing. The expression levels of lncRNAs in six samples were assessed by FPKM, and did not show a deviated distribution of mRNAs nor lncRNAs (**Figures 2A, B**). Scatterplots (**Figures 2C, D**) demonstrated high reproducibility of samples from each group.

**Figure 3:**
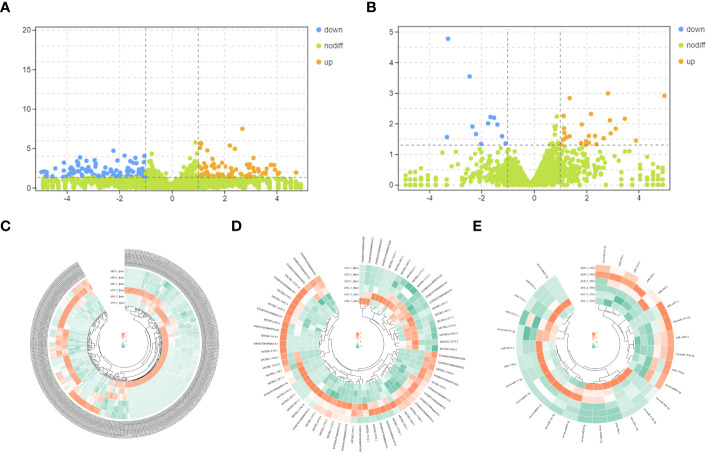
**(A)** Volcano plots of differentially expressed mRNAs. The abscissa represents the log of the multiple of the difference between the two groups, and the ordinate represents the negative Log10 value of the FDR of the difference between the two groups. **(B)** Volcano plots of differentially expressed lncRNAs. The abscissa represents the log of the multiple of the difference between the two groups, and the ordinate represents the negative Log10 value of the FDR of the difference between the two groups. **(C–E)** Heatmaps demonstrate the distribution of differentially expressed lncRNAs, miRNAs, and mRNAs.

Differentially expressed mRNAs and lncRNAs were analyzed using volcano plots (**Figures 3A, B**). Upregulated and downregulated mRNAs and lncRNAs, when compared to the geriatric NC group, were shown using orange and blue dots respectively. Ultimately, a total of 69, 364 and 29 differential expressed lncRNAs (49 up-regulated, 20 down-regulated), mRNAs (143 up-regulated, 221 down-regulated), and miRNAs (18 down-regulated, 11 up-regulated) respectively, were identified (**Figures 3C–E**).

### Validation of qPCR

**Figure 4:**
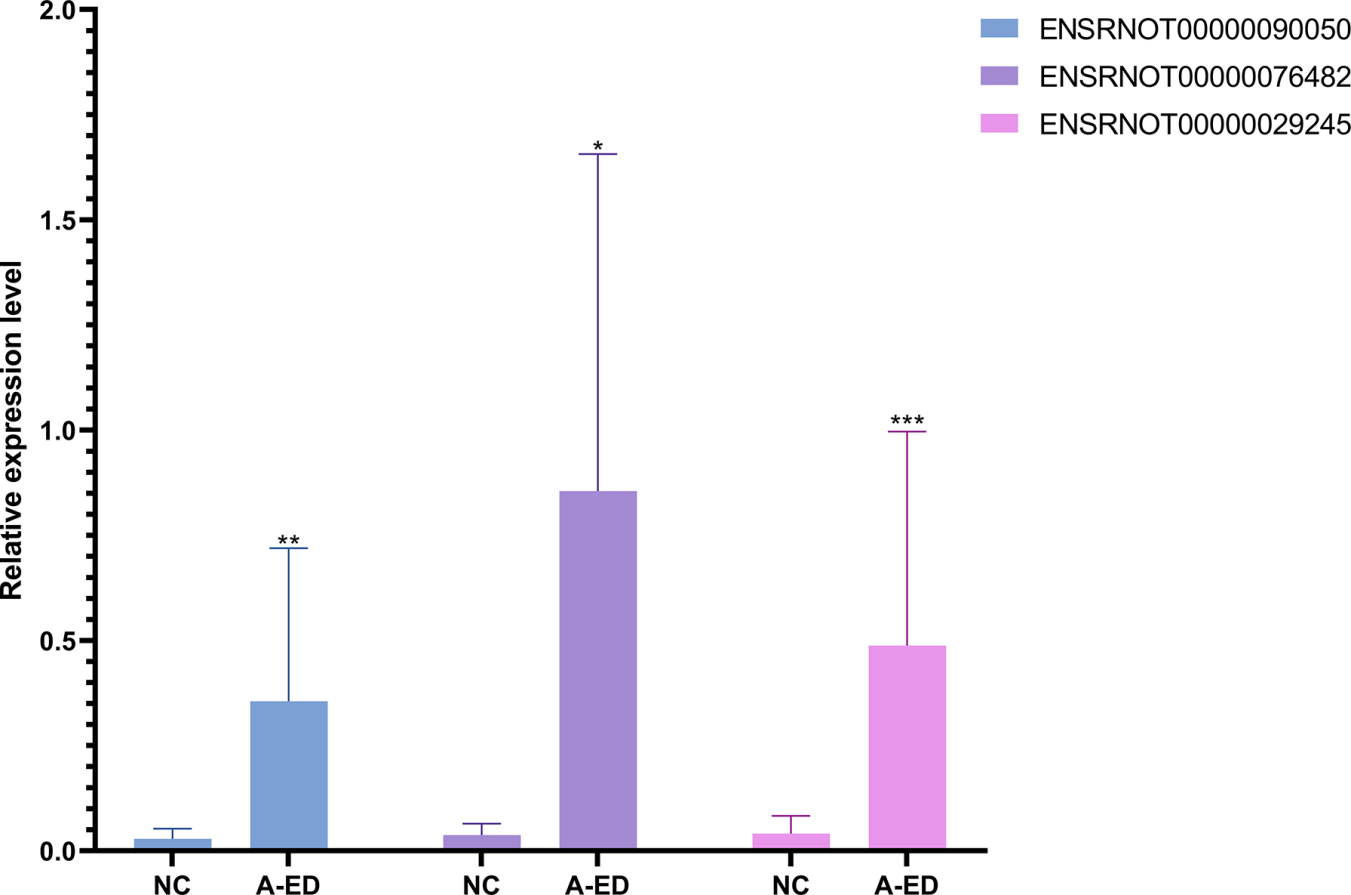
Bar cartoon of qPCR shows the expression of 3 lncRNAs between the A-ED and NC groups. *P < 0.05, **p < 0.01, ***p < 0.001.

Using an inclusion criteria for lncRNAs of 1<count number<120, fold change > 2, *p*< 0.05, seven lncRNAs were identified and used to validate RNA-seq results in seven pairs of geriatric ED and NC samples (MSTRG.3646.1, ENSRNOT00000085383, ENSRNOT00000093493, ENSRNOT00000081965, ENSRNOT00000029245, ENSRNOT00000090050, ENSRNOT00000076482, ENSRNOT00000085383). According to qPCR results, ENSRNOT00000076482(*p*=0.0175), ENSRNOT00000090050(*p*=0.007) and ENSRNOT00000029245(*p*=0.0006) were highly expressed in the ED group, corroborating sequencing results (**Figure 4**). However, expression levels of the other four lncRNAs did not statistical differ between groups.

### GO and KEGG Analysis

**Figure 5:**
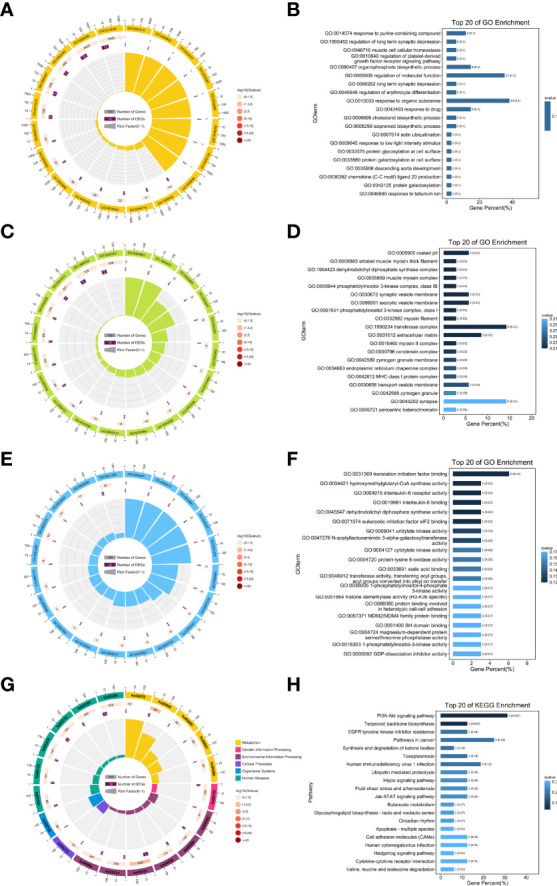
Results of the GO and KEGG enrichment. Top 20 enrichment results **(A, B)** biological processes; **(C, D)** cellular component; **(E, F)** molecular function; **(G, H)** KEGG pathways enrichment.

To further explore the specific function of these lncRNAs, related mRNAs were used to carry out GO and KEGG enrichment analyses and the top 20 results of enrichment analysis (including KEGG and GO) according to *p* values, are shown in **Figure 5**. In doing KEGG analyses, we identified pathways for PI3K-AKT & Jak-STAT signaling, terpenoid backbone biosynthesis, synthesis and degradation of ketone bodies, EGFR tyrosine kinase inhibitor resistance, fluid shear stress and atherosclerosis.

From GO enrichment analyses, cellular component (CC) annotations showed that the following components played key roles: coated pit (GO:0005905), striated muscle myosin thick filament (GO:0005863), dehydrodolichyl diphosphate synthase complex (GO:1904423), muscle myosin complex (GO:0005859), and phosphatidylinositol 3-kinase complex, class IB (GO:0005944). Corresponding annotations of molecular functions included translation initiation factor binding (GO:0031369), hydroxymethylglutaryl-CoA synthase activity (GO:0004421), and interleukin-6 receptor activity (GO:0004915).

Moreover, from the annotations of biological processes, the following were enriched (**Figure 5**): response to purine-containing compound (GO:0014074), regulation of long term synaptic depression (GO:1900452), muscle cell cellular homeostasis (GO:0046716), regulation of platelet-derived growth factor receptor signaling pathway (GO:0010640), and organophosphate biosynthetic process (GO:0090407).

### Construction of lncRNA-miRNA-mRNA Network

**Figure 6:**
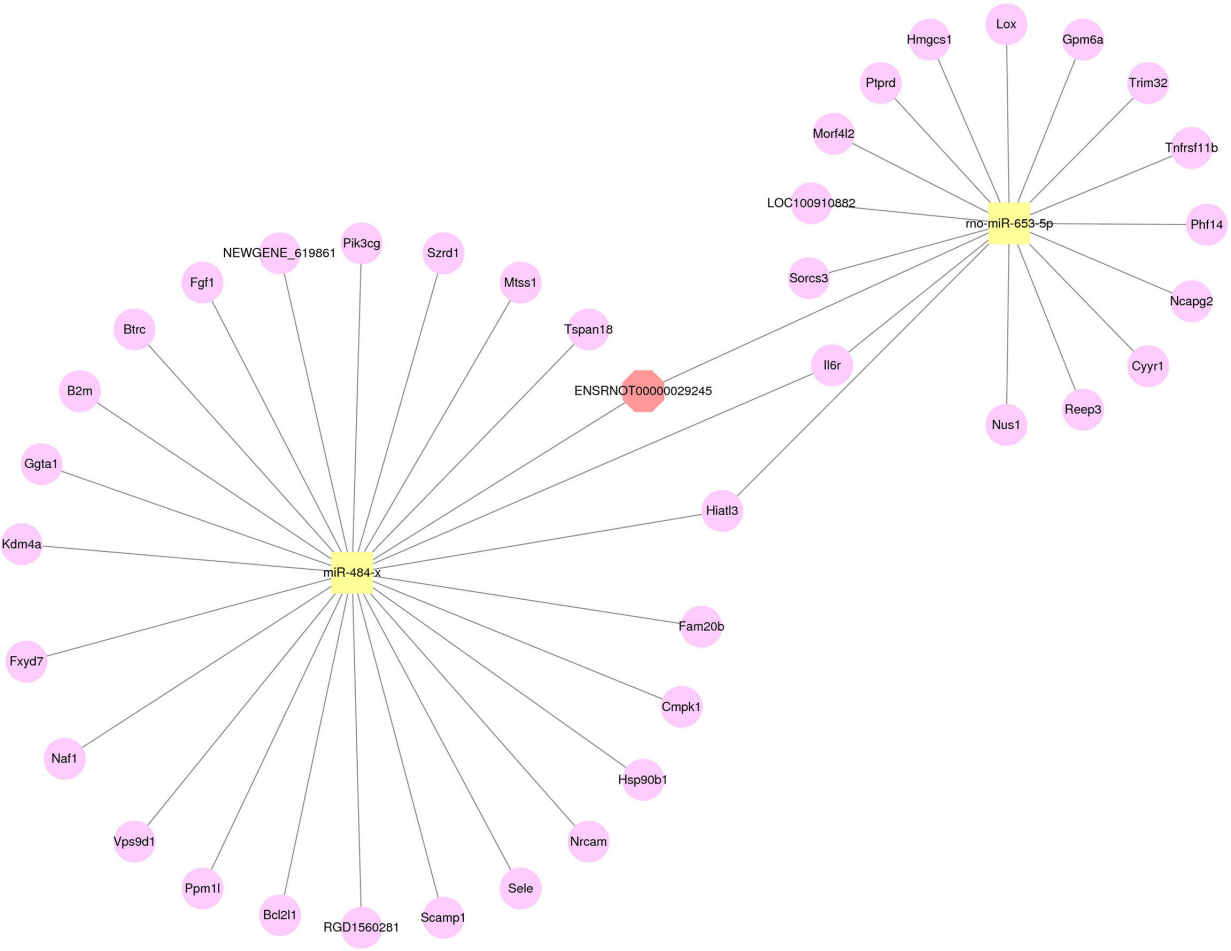
The network of lncRNA-miRNA-mRNA. Red octagons represent up-regulated lncRNAs, yellow squares represent up-regulated miRNAs, and pink circles represent up-regulated mRNAs.

From the results of RT-qPCR and RNA-seq, three verified lncRNAs, 29 differentially expressed miRNAs, and 364 differentially expressed mRNAs were selected, and the regulatory network was constructed according to its hypothesis. **Figure 6** illustrates the lncRNA-miRNA-mRNA regulatory network of geriatric ED rats. In our network, the up-regulated lncRNA ENSRNOT00000029245 upregulated the expression of 23 mRNAs (Tspan18, Mtss1, Szrd1, Pik3cg, NEWGENE_619861, Fgf1, Btrc, B2m, Ggta1, kdm4a, Fxyd7, Naf1, Vps9d1, Ppm1l, Bcl2l1, RGD1560281, Scamp1, Sele, Nrcam, Hsp90b1, Cmpk1, Fam20b, Hiatl3) by miR484x axis. Similarly, the lncRNA ENSRNOT00000029245 regulated the 14 mRNAs (Sorcs3, LOC100910882, Morf4l2, Ptprd, Hmgcs1, Lox, Gpm6a, Trim32, Tnfrsf11b, Phf14, Ncapg2, Cyyr1, Reep3, Nus1) by rno-miR-653-5p axis. Interestingly, according to our network, the mRNA Il6r was upregulated by both the miR484x axis and the rno-miR-653-5p axis.

### Validation in Cells

**Figure 7:**
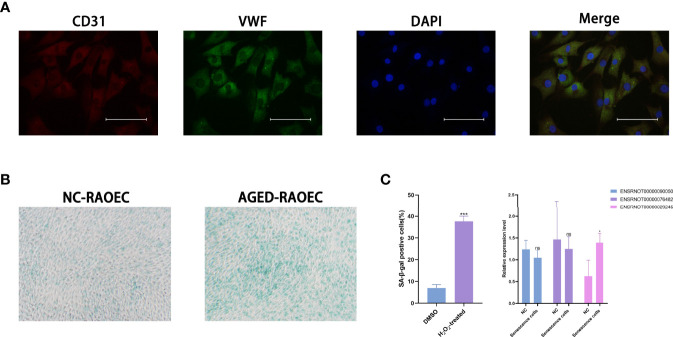
Cell lines validations. **(A)** Double-immunofluorescent staining for biomarkers CD31 and vWF. **(B)** β-Galactosidase staining for RAOEC cell in the DMSO and the H_2_O_2_ treated group. **(C)** Relative expression of three lncRNAs (ENSRNOT00000090050, ENSRNOT00000076482, and ENSRNOT00000029245) in Senescence cells and NC cells. *P < 0.05. Scale bars = 25 µm.

The RAOEC cells were identified by staining with anti-CD31 antibody (red), anti-vWF antibody (green), and cell nuclei were identified using DAPI (blue) (**Figure 7A**). The sa-β-Gal staining demonstrated that the RAOEC cells were induced senescence at 100μM H_2_O_2_ (**Figure 7B**). Interestingly, the expression of the three lncRNAs (ENSRNOT00000029245, ENSRNOT00000076482, and ENSRNOT00000090050) between cell lines showed that only ENSRNOT00000029245 had a statistical significance (**Figure 7C**).

## Discussion

As the global population of the elderly rises, so does erectile dysfunction, a disease closely related to aging. Physiologically, an erection occurs when, NO, released from the endothelium and parasympathetic nerve terminals, enhances cGMP, thus promoting the loss of intracellular calcium and resulting in the relaxation of smooth muscles. The venous return is then blocked, triggering an erection.

Although PDE5Is are currently recommended as first-line treatments, they only relieve symptoms without curing, and the effective PDE5 dose increases with age (26). This, therefore, necessitates new insights into the molecular biology of age-related erectile dysfunction for the discovery of novel drug targets and therapies.

In this study, we comparatively analyzed transcriptomes based on a *p*< 0.05, logFC>2 criteria, to identify differentially expressed RNAs. A total of 69, 364, and 29 differentially expressed lncRNAs, mRNAs, and miRNAs respectively, were identified from comparisons of transcriptomes of geriatric ED and NC rats. Using the criteria of 1<count number<120, seven lncRNAs were identified and validated by qPCR. Furthermore, three lncRNAs were differentially expressed between the two groups. However, only lncRNA ENSRNOT00000029245 was used to successfully construct a lncRNA-miRNA-mRNA regulatory network.

Moreover, we conducted GO and KEGG enrichment analysis based on lncRNA ENSRNOT00000029245 related mRNAs. The results of these enrichment analyses showed that in terms of GO enrichment, coated pit, striated muscle myosin thick filament, dehydrodolichyl diphosphate synthase complex, muscle myosin complex, phosphatidylinositol 3-kinase complex, and translation initiation factor binding were enriched. The results of the KEGG pathway analysis indicated that PI3K-AKT and Jak-STAT signaling, terpenoid backbone biosynthesis, synthesis and degradation of ketone bodies, EGFR tyrosine kinase inhibitor resistance, fluid shear stress, and atherosclerosis pathways were enriched. The PI3K-AKT signaling pathway plays a critical role in maintaining vascular endothelial function (27). It also regulates the opening of the endothelial tight junction, as well as the permeability and mobility of endothelial cells (28). This suggests that aging leads to mutations in the PI3K pathway, which in turn leads to erectile dysfunction by affecting endothelial cell function.

ceRNA crosstalk has possible roles in numerous diseases, as lncRNAs regulate diseases through ceRNA (29, 30). Using both an exclusion criteria and qPCR results, we elected the differentially expressed lncRNA ENSRNOT00000029245 to build a lncRNA-miRNA-mRNA regulate network that could identify microregulatory processes in age-related ED.

Phosphatidylinositol 3-kinase (PI3K), an important component in the PI3K-AKT pathway, is a vascular protective factor with anti-apoptotic functions (31). Yu et al. found that PI3Kγ in vascular smooth muscle cells(VSMC) was upregulated after vascular injury, and PI3Kγ could promote proliferation and migration of VSMC (32). Thus, PI3Ks possibly contribute in large part to ED. In our study, ENSRNOT00000029245 was validated by qPCR as highly expressed in ED rats, and related to Pik3cg. In our regulatory network, ENSRNOT00000029245 regulates Pik3cg by modulating miR484-x. High expression of miR-484 downregulates eNOS transcripts, thus affecting homeostasis of endothelial function (33).

ED is accompanied by irreversible death of smooth muscle and endothelial cells (2). BCL2 family proteins are important regulators of apoptosis. Our network indicates that LncRNA ENSRNOT00000029245 may be associated with apoptosis in either cavernosal endothelium or smooth muscle cells *via* regulating miR-484-x-mediated Bcl2l1 expression. Moreover, tripartite motif-containing 32 (trim32), is up-regulated when cells undergo oxidative stress, promote ROS generation, and induce apoptosis (34). From our findings, trim32 was upregulated *via* the ENSRNOT00000029245-rno-miR-653-5p axis. This supported the hypothesis that upregulated ENSRNOT00000029245 promotes apoptosis of corporeal smooth muscle and endothelial cells, thus inducing ED in geriatric rats.

Our network findings suggest that ENSRNOT00000029245 targets miRNAs and mRNAs that affect either fibrosis or apoptosis in the corpus cavernosum and this ultimately results in erectile function.

However, our study still has some weaknesses. First, our model was based on rats and, despite lncRNAs being conserved between humans and rats, it thus does not provide direct evidence for the feasibility of our ceRNA network for humans. Ideally, this network should have been constructed from comparisons of cavernous tissues of elderly-ED patients to that of elderly healthy volunteers. Second, we constructed the ceRNA network of lncRNA without considering crosstalk from pseudogenes and circular RNAs. Lastly, we did not validate the proposed regulatory effect of this lncRNA on erectile function *in vivo*.

## Conclusion

In this study, we identified 69, 29, and 364 differentially expressed lncRNAs, miRNAs, and mRNAs respectively, by comparing transcriptomes from A-ED rats to those from NC rats. Three lncRNAs (ENSRNOT00000029245, ENSRNOT00000076482, and ENSRNOT00000090050) were validated by qPCR, as highly expressed in the A-ED group. The results of GO enrichment and KEGG pathway analyses showed that A-ED may be associated with PI3K-AKT signaling, fluid shear stress, and atherosclerosis pathways. Moreover, the constructed network showed that ENSRNOT00000029245 possibly contributes to either fibrosis or apoptosis in the corpus cavernosum *via* either a rno-miR-653-5p-trim32 axis or by regulating miR-484-x-mediated Bcl2l1 expression. Therefore, it is worthwhile to investigate molecular mechanisms through which lncRNA ENSRNOT00000029245 regulates age-related ED.

## Data Availability Statement

The RNA-Seq data presented in the study are deposited in the SRA databank (www.ncbi.nlm.nih.gov/sra) and is accessible via the PRJNA848782 SRA (Bioproject) accession number.

## Ethics Statement

The animal study was reviewed and approved by The Animal Care and Use Committee of the First Affiliated Hospital of Nanjing Medical University.

## Author Contributions

NS and XM designed this work. XM, XZ (1^st^ author), LY, and RC wrote the manuscript. QZ, XiZ, and JL performed the bioinformatics analysis. XZ (7^th^ author), XR, and TZ performed the data review. All authors have read and approved the manuscript.

## Funding

This article was funded by the National Natural Science Foundation of China [grant number: 81801438; 81871151].

## Conflict of Interest

The authors declare that the research was conducted in the absence of any commercial or financial relationships that could be construed as a potential conflict of interest.

## Publisher’s Note

All claims expressed in this article are solely those of the authors and do not necessarily represent those of their affiliated organizations, or those of the publisher, the editors and the reviewers. Any product that may be evaluated in this article, or claim that may be made by its manufacturer, is not guaranteed or endorsed by the publisher.

## Supplementary Material

Supplementary Figure 1 The bar plot of qPCR of these 7 lncRNAs between A-ED and NC group (*p < 0.05, **p < 0.01, ***p < 0.001).

## Abbreviations

ED, Erectile Dysfunction; A-ED, Age-related Erectile dysfunction; PDE5-Is, phosphodiesterase type 5 inhibitors; GO, Gene ontology; KEGG, Kyoto Encyclopedia of Genes and Genomes; qPCR, Quantitative real-time PCR; MAP, Mean arterial pressure; ICP, Intracavernous pressure; lncRNAs, Long non-coding ribonucleic acids.
