# Supplementary figures and images for: Comparative Transcriptome Analyses of Geriatric Rats Associate Age-Related Erectile Dysfunction With a lncRNA-miRNA-mRNA Regulatory Network
Source: Front Endocrinol (Lausanne). 2022 Jul 11;13:887486. doi: 10.3389/fendo.2022.887486 (PMC9309694; doi:10.3389/fendo.2022.887486)

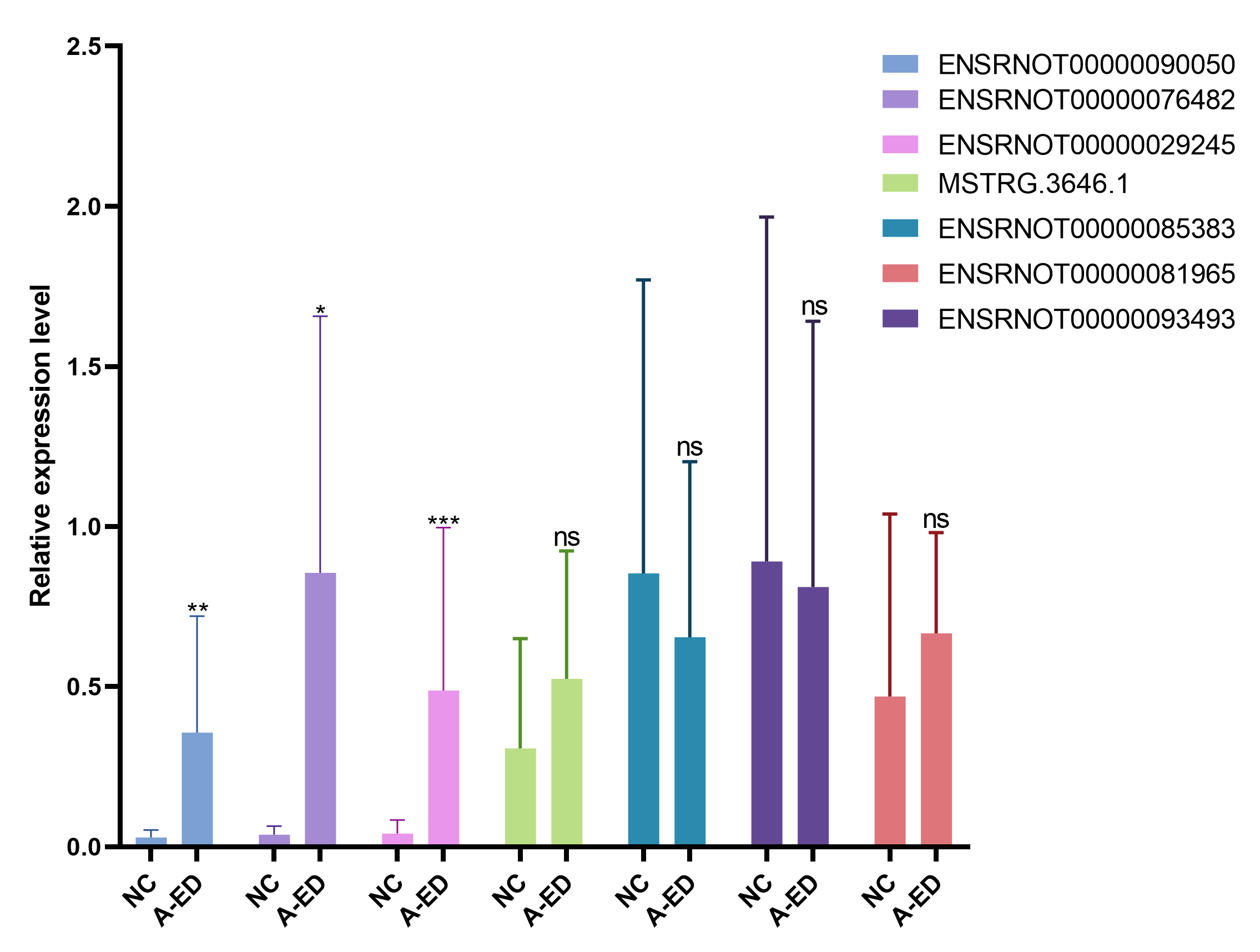

Supplement: Supplementary Figure 1 — The bar plot of qPCR of these 7 lncRNAs between A-ED and NC group (*p < 0.05, **p < 0.01, ***p < 0.001). [file Image_1.tif]
