# Supplementary material for: Comparative Transcriptome Analyses of Geriatric Rats Associate Age-Related Erectile Dysfunction With a lncRNA-miRNA-mRNA Regulatory Network
Source: Front Endocrinol (Lausanne). 2022 Jul 11;13:887486. doi: 10.3389/fendo.2022.887486 (PMC9309694; doi:10.3389/fendo.2022.887486)
Supplement: Supplementary file 2 [file Table_1.docx]

**Supplementary Table S1 Oligonucleotide sequences used in this study**

| Primes and probes | Forword Sequences | Reverse Sequences |
| --- | --- | --- |
| MSTRG.3646.1 | CCGACAGAGAGAGCAAGCAA | CCCTTGGCATGAGGTTGAGT |
| ENSRNOT00000085383 | GGCATCGTTGAAGACTGGGA | TACAGTAGCCACCCCTCCTC |
| ENSRNOT00000093493 | CTGAAACTGGGAAAGGG | TCTGAAGGGCTTGGGAT |
| ENSRNOT00000081965 | AAGGCCAGAGGACTAAA | AGATGGAGGCATACACG |
| ENSRNOT00000029245 | TGCTGAGGCTCTGTTGG | TGAGCGTGGTAGGTGGC |
| ENSRNOT00000090050 | ACAGCGGAACTCAAGAC | TGACTGAAAGAGCGAGAC |
| ENSRNOT00000076482 | TAATGTGGTTGCCGATGA | CTGGGTGTAAATGCTTCG |
| ENSRNOT00000085383 | GGCATCGTTGAAGACTGGGA | TACAGTAGCCACCCCTCCTC |
